# Outbreak of Influenza A (H3N2) Variant Virus Infection among Attendees of an Agricultural Fair, Pennsylvania, USA, 2011

**DOI:** 10.3201/eid1812.121097

**Published:** 2012-12

**Authors:** Karen K. Wong, Adena Greenbaum, Maria E. Moll, James Lando, Erin L. Moore, Rahul Ganatra, Matthew Biggerstaff, Eugene Lam, Erica E. Smith, Aaron D. Storms, Jeffrey R. Miller, Virginia Dato, Kumar Nalluswami, Atmaram Nambiar, Sharon A. Silvestri, James R. Lute, Stephen Ostroff, Kathy Hancock, Alicia Branch, Susan C. Trock, Alexander Klimov, Bo Shu, Lynnette Brammer, Scott Epperson, Lyn Finelli, Michael A. Jhung

**Affiliations:** Centers for Disease Control and Prevention, Atlanta, Georgia, USA (K.K. Wong, A. Greenbaum, R. Ganatra, M. Biggerstaff, E. Lam, A.D. Storms, J.R. Miller, K. Hancock, A. Branch, S.C. Trock, A. Klimov, B. Shu, L. Brammer, S. Epperson, L. Finelli, M.A. Jhung);; Pennsylvania Department of Health, Harrisburg, Pennsylvania, USA (M.E. Moll, E.E. Smith, J.R. Miller, V. Dato, K. Nalluswami, A. Nambiar, J.R. Lute, S. Ostroff);; Allegheny County Health Department, Pittsburgh, Pennsylvania (J. Lando, S.A. Silvestri);; and Pennsylvania Department of Agriculture, Harrisburg (E.L. Moore)

**Keywords:** influenza, human, influenza A virus, H3N2 subtype, H3N2 subtype variant, swine diseases, zoonoses, viruses, Pennsylvania, transmission, swine, agriculture

## Abstract

During August 2011, influenza A (H3N2) variant [A(H3N2)v] virus infection developed in a child who attended an agricultural fair in Pennsylvania, USA; the virus resulted from reassortment of a swine influenza virus with influenza A(H1N1)pdm09. We interviewed fair attendees and conducted a retrospective cohort study among members of an agricultural club who attended the fair. Probable and confirmed cases of A(H3N2)v virus infection were defined by serology and genomic sequencing results, respectively. We identified 82 suspected, 4 probable, and 3 confirmed case-patients who attended the fair. Among 127 cohort study members, the risk for suspected case status increased as swine exposure increased from none (4%; referent) to visiting swine exhibits (8%; relative risk 2.1; 95% CI 0.2–53.4) to touching swine (16%; relative risk 4.4; 95% CI 0.8–116.3). Fairs may be venues for zoonotic transmission of viruses with epidemic potential; thus, health officials should investigate respiratory illness outbreaks associated with agricultural events.

## Abstract 2

Avoiding or limiting contact with swine at agricultural events may help prevent A(H3N2)v virus infections in such settings.

Triple reassortant swine influenza A viruses have circulated in swine herds in North America since 1998 (*1*–*3*). On the rare occasions that these viruses infect humans, they are called influenza A variant viruses (*4*). Viruses resulting from reassortment of swine influenza A (H3N2) virus and influenza A(H1N1)pdm09 (pH1N1) virus have emerged among US swine (*4*–*6*), and similar viruses have been identified among swine outside the United States (*7*,*8*). During August 2011, the first known human infection with influenza A (H3N2) variant [A(H3N2)v] virus containing the pH1N1 matrix (M) gene was reported in the United States (*9*). The pH1N1 M gene is implicated in increasing influenza transmissibility in animal models (*10*,*11*), and there was concern that this new A(H3N2)v virus could be efficiently transmitted among humans. Because these viruses contain a novel combination of genes, little is known about the epidemiologic and clinical characteristics of human infections.

During August 2011, a child who had attended an agricultural fair in Pennsylvania (Fair A) attended by ≈70,000 persons became ill; the Centers for Disease Control and Prevention (CDC) confirmed infection with A(H3N2)v virus in the child 6 days after Fair A closed and immediately began an investigation with the Pennsylvania Department of Health (PA DOH), the Allegheny County Health Department, and the Pennsylvania Department of Agriculture (PDA) to determine the extent of A(H3N2)v virus transmission and to identify illness risk factors among Fair A attendees.

## Methods

### Case Finding

We identified cases through multiple methods. First, 1–2 weeks after Fair A closed, PDA investigators conducted open-ended interviews with swine exhibitors to determine whether they or their household members had become ill; exhibitors were identified though a list provided by fair organizers. Ill exhibitors or their surrogates were interviewed by CDC/PA DOH to assess whether their illness met suspected case criteria. Second, members of a national children’s agricultural club who participated in activities in the county where Fair A occurred were interviewed about illness occurring in their households after attendance at Fair A and/or swine exposure. Third, investigators went to another fair (Fair B), which occurred 3 weeks after Fair A ≈20 miles away in the same county, to enroll a convenience sample of Fair B attendees. Investigators enrolled Fair B attendees as they visited the health department booth, food service areas, exhibit halls, rides, and games. Fair B enrollees were subsequently asked during a phone interview whether they or their household members had become ill after attending Fair A. Fourth, media sources, including newspapers, television, and websites, encouraged community members to contact PA DOH if they became ill with influenza-like symptoms after attending Fair A. Fifth, clinicians were encouraged to obtain respiratory specimens from patients with suspected influenza virus infection who had recent swine or agricultural fair exposure and to refer such patients to PA DOH for interview. Sixth, state and county influenza surveillance supported prospective detection of persons with test results positive for influenza. Seventh, ill contacts of case-patients were interviewed by investigators.

If close contacts of case-patients became ill, they were offered testing for influenza virus infection regardless of whether they attended Fair A. Testing was done only if specimens could be collected <7 days after symptom onset; nasopharyngeal swab samples were used for testing.

### Retrospective Cohort Study

We conducted a retrospective cohort study among a systematic random sample of members of a children’s agricultural club (Club X) who resided in the county where Fair A occurred and who attended Fair A. Club members were children who conducted projects, such as raising livestock, for exhibition at Fair A. From a list of 994 Club X members, every fourth name was selected, yielding a cohort of 247 children. Using a standard questionnaire, we queried the parents of Club X members about illness occurring since Fair A; animal exposures at Fair A, home, work, and/or school; influenza vaccination history; and underlying medical conditions. If no adult was reached after 3 telephone attempts, the household members were considered nonrespondents. Interviews were conducted 3–4 weeks after Fair A concluded. Levels of swine exposure were categorized as 1) no exposure (attending Fair A but not visiting a swine exhibit); 2) indirect exposure (visiting a swine exhibit but not touching swine at Fair A); and 3) direct exposure (touching swine at Fair A). Risk for illness was estimated from the beginning of Fair A through 7 days after its conclusion.

### Case Definitions

A suspected case-patient was a person with >1 sign/symptom of influenza virus infection from >2 of 4 categories occurring <7 days after attending Fair A. Categories were: 1) fever (temperature >38C°) or subjective fever; 2) respiratory (cough, sore throat, or runny nose); 3) gastrointestinal (vomiting or diarrhea); and 4) constitutional (fatigue, muscle aches, or joint pain). At least 1 category was required to be fever or respiratory.

A broad clinical case definition was used because the clinical characteristics of A(H3N2)v virus infections were not well understood. Suspected case-patients were reclassified as noncase-patients if respiratory specimens obtained <7 days after symptom onset had real-time reverse transcription PCR (rRT-PCR) or genomic sequencing results negative for A(H3N2)v virus or if convalescent-phase serology results were negative for A(H3N2)v virus infection.

A probable case-patient was a person <4 years of age (explained below) who met suspected case-patient criteria and who was seropositive for A(H3N2)v virus. A confirmed case-patient was a person who had rRT-PCR and genomic sequencing results positive for A(H3N2)v virus infection; RNA from a respiratory specimen was used for genomic sequencing (*12*).

### Influenza Diagnostic Testing

#### Respiratory Specimens

Respiratory specimens were obtained <7 days after symptom onset. We used rRT-PCR with the Human Influenza Virus Real-Time RT-PCR Diagnostic Panel (CDC, Atlanta, GA, USA) to test specimens. Specimens positive for influenza A were subtyped, and amplified RNA from specimens with results consistent with A(H3N2)v virus infection (positive for InfA, pdmInfA, and H3 markers) or with indeterminate results underwent partial genome sequencing as described (*12*,*13*).

#### Serologic Testing

We asked suspected case-patients <13 years of age to participate in serologic testing. We chose this age group for testing because it is assumed that children have limited prior exposure to viruses similar to A(H3N2)v virus and therefore fewer cross-reactive antibodies. A convalescent-phase serum sample was obtained from participating suspected case-patients 3–5 weeks after illness onset. Serum samples were tested by microneutralization and hemagglutination inhibition (HI) for antibodies to variant strains A/Minnesota/11/2010 (H3N2) and A/Indiana/08/2011 (H3N2). The outbreak strain could not be used as an antigen because viable virus was not isolated from any of the case-patients. Microneutralization and HI tests were performed as described (*14*).

Preliminary testing of serum samples collected in 2007–2008 and in 2010 indicates that no children <4 years of age have antibodies to A(H3N2)v virus, but some children 4–13 years of age have cross-reactive antibodies (*15*). Therefore, test results for children <4 years of age were considered seronegative if HI titers to the variant strains were <10, indeterminate if titers were 10 to <40, and seropositive if titers were >40. Test results for children 4–13 years of age were considered seronegative if titers to the variant strains were <10 and indeterminate if titers were >10. Children with seronegative test results were reclassified as noncase-patients, and those with seropositive results were reclassified as probable case-patients. Children with indeterminate results retained suspected case-patient status.

### Animal Investigation

PDA veterinarians routinely inspected all swine at Fair A. In addition, veterinarians called Fair A swine exhibitors 1–2 weeks after Fair A closed to ask whether signs of illness developed in any swine during or shortly after Fair A.

### Data Analysis

We entered data into a Microsoft Access 2010 database (Microsoft, Redmond, WA, USA) and analyzed it by using SAS version 9.3 (SAS Institute, Cary, NC, USA). Relative risks and exact 95% CIs, determined by using the Farrington-Manning method (*16*), are reported for selected exposures.

### Ethical Considerations

This investigation was determined to be a response to a public health threat; in accordance with Federal human subjects’ protection regulations, it was not considered to be human subjects research. Participation in interviews was voluntary; parents or guardians were interviewed for subjects <18 years of age. Parents or guardians consented to collection of respiratory and serum samples from subjects <18 years of age. Minors >7 years of age assented to collection of respiratory and serum samples.

## Results

### Case Finding

We identified 3 confirmed, 4 probable, and 82 suspected cases, including the index case. No A(H3N2)v virus infections were identified by state or county influenza surveillance or by clinicians among persons who did not attend Fair A. Of the confirmed, probable, and suspected cases, 19 (21%) were identified from Fair A swine exhibitor households, 29 (33%) were identified from Club X households, 4 (4%) were identified among Fair B attendees who also attended Fair A, 34 (38%) were identified among persons who called PA DOH to report illness, 10 (11%) were identified by another case-patient, and 2 (2%) were detected by state influenza surveillance; persons could be identified by >1 method. The median age of all case-patients was 12 years (range 6 months–60 years); 39 (44%) were male (Table 1). Dates of illness onset ranged from day 0–13, where day 0 was the opening day for Fair A (Figure). Most case-patients had illness onset within 4 days after either the swine show or swine auction, and no cases were identified >6 days after the fair ended. Of 87 case-patients for whom medical history was known, 18 (21%) reported at least 1 underlying medical condition. Case-patients reported spending a median of 6 days (range 1–10 days) at Fair A, and 29 (33%) of 89 reported that their household owned swine. Of 87 case-patients for whom swine exposure was known, 80 (92%) reported direct or indirect swine exposure at Fair A.

**Table 1:** Characteristics of case-patients with suspected, probable, or confirmed A(H3N2)v virus infection after attending agricultural Fair A, Pennsylvania, 2011*

Characteristic	A(H3N2)v case status		
	Suspected, n = 82	Probable and confirmed, n = 7	All cases, N = 89
Sex			
M	37 (45)	2 (29)	39 (44)
F	45 (55)	5 (71)	50 (56)
Age, y			
Median (range)	13 (0.5–60)	3 (1–9)	12 (0.5–60)
<4	9 (11)	5 (71)	14 (16)
4–13	34 (41)	2 (29)	36 (40)
14–18	12 (15)	0	12 (13)
19–49	17 (21)	0	17 (19)
>50	10 (12)	0	10 (11)
Underlying medical condition†	17/80 (21)	1/7 (14)	18/87 (21)
Reported any prior influenza vaccination	49/73 (67)	5/5 (100)	54/78 (69)
No. days spent at fair, median (range)	7 (1–10)	4 (1–8)	6 (1–10)
Family owns swine	27/82 (33)	2/7 (29)	29/89 (33)
Reported swine exposure‡	73/80 (91)	7/7 (100)	80/87 (92)
Signs/symptoms			
Fever/feverish	74/82 (90)	7/7 (100)	81/89 (91)
Cough	58/80 (73)	6/7 (86)	64/87 (74)
Runny nose	38/77 (49)	3/7 (43)	41/84 (49)
Headache	58/72 (81)	0/4	58/76 (76)
Muscle aches	40/69 (58)	0/5	40/74 (54)
Sore throat	44/72 (61)	1/5 (20)	45/77 (58)
Shortness of breath	16/69 (23)	1/7 (14)	17/76 (22)
Vomiting	21/82 (26)	2/7 (29)	23/89 (26)
Diarrhea	27/82 (33)	2/7 (29)	29/89 (33)
Fatigue	55/57 (96)	3/3 (100)	58/60 (97)
Joint pain	18/53 (34)	0/1	18/54 (33)
Illness duration, median days (range)	7 (2–18)§	6 (3–9)¶	6 (2–18)**
Received medical attention	28/81 (35)	3/7 (43)	31/88 (35)
Hospitalized	0/81	1/7 (14)	1/88 (1)

*Data are no. (%) with characteristic or no. with characteristic/total no. case-patients with data (%) unless otherwise specified. A(H3N2)v, influenza A (H3N2) variant virus. †Includes asthma, chronic lung disease, heart disease, diabetes or metabolic disease, kidney disease, liver disease, immunosuppressive condition, cancer, and neurologic or neurodevelopmental disorders. ‡Includes visiting swine exhibit, walking near swine exhibit, or touching swine at fair. §Of 60 persons with known dates of illness onset and recovery. ¶Of 5 persons with known date of recovery. **Of 65 persons with known date of recovery.

**Figure:**
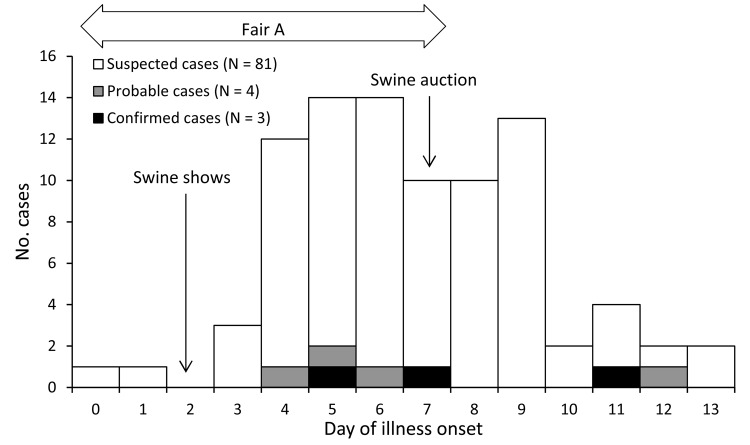
Epidemic curve, by date of illness onset and case status, for 88 cases of influenza A (H3N2) variant virus infection associated with agricultural Fair A, Pennsylvania, 2011. Day 0 is the first day the fair was open to the public. One suspected case is not shown; the day of illness onset is unknown but <7 days after attending the fair.

The first confirmed case occurred in a previously healthy girl <4 years of age who touched swine at Fair A (case-patient 1); fever, cough, and rhinorrhea developed 4 days after she had contact with swine. The second confirmed case occurred in a previously healthy girl in the 4- to 13-year-old age group who exhibited swine at Fair A and had subjective fever and vomiting without respiratory symptoms (case-patient 2). The third confirmed case occurred in a girl in the 4- to 13-year-old age group who had a preexisting medical condition (case-patient 3); the girl had contact with swine at Fair A and was hospitalized for respiratory distress. Of 3 confirmed and 4 probable case-patients, 2 (29%) were male and all were <13 years of age. All confirmed and probable case-patients attended Fair A on or after day 3 of the fair (Table 2). All except case-patient 2 had fever and respiratory symptoms, and all recovered. All 7 confirmed and probable case-patients visited the swine exhibit at Fair A, and 6 (86%) touched swine.

**Table 2:** Characteristics of case-patients with confirmed or probable A(H3N2)v virus infection after attending an agricultural Fair A, Pennsylvania, 2011*

Case-patient no., case status	Age group, y/sex	Underlying condition	Date(s) at fair†	Date of illness onset‡	Symptoms	Clinical outcome
1, confirmed	<4/F	No	3	7	Fever, cough, runny nose	Visited clinic, recovered
2, confirmed	4–13/F	No	0 to 7	11	Feverish, vomiting, abdominal pain	Did not seek medical care, recovered
3, confirmed	4–13/F	Yes	2 to 5	5	Fever, cough, dyspnea, vomiting, diarrhea	Hospitalized, ICU (not mechanically ventilated), recovered
4, probable	<4/M	No	4	6	Fever, cough, runny nose, diarrhea	Visited clinic, recovered
5, probable	<4/F	No	0 to 3, 5, 6	5	Fever, cough	Did not seek medical care, recovered
6, probable	<4/M	No	6	12	Fever, cough, sore throat, runny nose	Did not seek medical care, recovered
7, probable	<4/F	No	−1§ to 2, 4, 5, 7	4	Feverish, cough	Did not seek medical care, recovered

*Case-patients 1–7 visited the swine exhibit, and all except case-patient 4 also touched swine. Case-patient 2 exhibited swine with an agricultural club for children (Club X) and was not randomly selected for the cohort study. A(H3N2)v, influenza A (H3N2) variant virus; ICU, intensive care unit. †Day 0 is the first day fair was open to the public. ‡Illness onset date represents number of days after fair was open to the public. §Arrived at fairgrounds 1 day before fair was open to the public.

Illness developed in contacts of 4 case-patients (3 suspected and 1 confirmed case-patient); the contacts had not attended Fair A <7 days before illness onset. Respiratory specimens were obtained from 3 of these 4 contacts, including the contact of the confirmed case-patient, <7 days after illness onset; all tested negative for influenza by rRT-PCR, and 1 contact tested positive for rhinovirus. One person declined testing for influenza.

### Laboratory Results

Respiratory specimens from case-patients 1 and 3 were positive for InfA, H3, and pdmInfA markers by rRT-PCR, and the specimen from case-patient 2 was InfA positive. Phylogenetic analysis of the 3 specimens showed that the genome contained the M gene from pH1N1 and 7 gene segments (hemagglutinin, neuraminidase, polymerase PB1, polymerase PB2, polymerase PA, nucleocapsid protein, nonstructural protein) similar to those from North American swine H3N2 subtype viruses and variant viruses that previously caused infection in humans (*13*).

Convalescent-phase serum samples were obtained from 6 (40%) of 15 persons <4 years of age who initially met suspected case-patient criteria; 4 (67%) of the 6 samples were seropositive for A(H3N2)v virus (HI geometric mean titers >57), and 2 (33%) were seronegative. Convalescent-phase serum samples were obtained from 18 (47%) of 38 persons 4–13 years of age who initially met suspected case-patient criteria; 4 (22%) of the 18 samples were seronegative for A(H3N2)v, and 14 (78%) had indeterminate results.

### Retrospective Cohort Study

We were able to contact 139 (56%) of the 247 Club X members; 127 (91%) of those contacted agreed to be interviewed. The median age of Club X members was 13 years (range 4–19 years); 47 (37%) were male (Table 3). Of 124 members, 19 (15%) reported >1 underlying medical condition. Members spent a median of 9 days at the fair (range 1–10 days), and 75/125 (60%) exhibited animals at Fair A; 34/125 (27%) exhibited swine. Of 125 families, 83 (66%) owned livestock and 33 (26%) owned swine.

**Table 3:** Characteristics of Club X cohort members who attended agricultural Fair A during an outbreak of A(H3N2)v virus infection, Pennsylvania, 2011*

Characteristic	Club X cohort, N = 127
Sex	
M	47/127 (37)
F	80/127 (63)
Case status	
Suspected case-patient	14/127 (11)
Non–case-patient	113/127 (89)
Age, y	
Median (range)	13 (4–19)
<4 y	2/127 (2)
4–13	70/127 (55)
14–18	51/127 (40)
>19	4/127 (3)
Underlying medical condition	19/124 (15)
Reported any prior influenza vaccination	56/115 (49)
No. days spent at fair, median (range)	9 (1–10)
Exhibited animal at fair	75/125 (60)
Exhibited swine at fair	34/125 (27)
Family owns livestock	83/125 (66)
Family owns swine	33/125 (26)
Reported swine exposure†	100/127 (79)

*Data are no. with characteristic (%) or no. with characteristic/total no. cohort members with data (%) unless otherwise specified. Club X is an agricultural club for children. A(H3N2)v, influenza A (H3N2) variant virus. †Includes attending swine exhibit, walking near swine exhibit, or touching swine at fair.

Of 127 Club X members, 15 initially met the suspected case definition. Serologic testing was performed for 3 members: 1 was seronegative for A(H3N2)v virus and was reclassified as a noncase-patient, and 2 had indeterminate results. Thus, 14 (11%) of the 127 Club X members were suspected case-patients. Respiratory specimens were not obtained from any club members.

The risk for suspected case status increased as exposure to swine increased from no exposure (referent) to indirect exposure (relative risk [RR] 2.1; 95% CI 0.2–53.4) to direct exposure (RR 4.4; 95% CI 0.8–116.3; p = 0.07 by Cochran-Armitage trend test); however, these differences were not statistically significant (Table 4). Exhibiting swine was not associated with suspected case-patient status (RR 1.1; 95% CI 0.2–3.2). Suspected case-patient status was more common, but not statistically significantly so, among persons whose families owned swine and among persons who fed or bathed swine or who cleaned the swine pen during the fair (Table 4).

**Table 4:** Characteristics of Club X cohort members, by case status, who attended agricultural Fair A during an outbreak of A(H3N2)v virus infection, Pennsylvania, 2011*

Characteristic	No. members, N = 127	No. (%) suspected cases	RR† (95% CI)
Sex			
M	47	5 (11)	
F	80	9 (11)	
Age, y			
<4	2	0 (0)	
4–13	70	7 (10)	
14–18	51	7 (14)	
>19	4	0 (0)	
Underlying medical condition			
No	105	14 (13)	
Yes	19	0 (0)	
Swine exposure‡			
None	27	1 (4)	Referent
Indirect	39	3 (8)	2.1 (0.2–53.4)
Direct	61	10 (16)	4.4 (0.8–116.3)
Exhibited swine			
No	91	10 (11)	
Yes	34	4 (12)	1.1 (0.2–3.2)
Family owns swine			
No	92	7 (8)	
Yes	33	7 (21)	2.8 (0.9–8.6)
Fed swine			
No	78	7 (9)	
Yes	37	7 (19)	2.1 (0.7–6.6)
Bathed swine			
No	80	7 (9)	
Yes	35	7 (20)	2.3 (0.7–7.1)
Cleaned swine pen			
No	84	7 (8)	
Yes	31	7 (23)	2.7 (0.8–8.3)

*Club X is an agricultural club for children. A(H3N2)v, influenza A (H3N2) variant virus. RR, relative risk. †RR of suspected case status is reported for selected exposures. ‡Direct exposure defined as touching pigs; Indirect exposure defined as attending or walking near the pig exhibit.

### Animal Investigation

The PDA veterinarian inspected >150 swine on day 3 of Fair A. All swine were healthy-appearing at inspection, although fever had developed in 1 pig, and that pig had already been removed from the fair. The febrile pig was housed with other swine at the fair for ≈24 hours before removal; it was not tested for influenza. Another pig died after a seizure on the last day of the fair; the cause of death was unknown.

No other illness in swine was reported to PDA during the fair. After the fair ended, PDA veterinarians attempted to call 135 households of swine exhibitors and reached 80 (59%). Of those 80 households, 8 (10%) reported that the swine they exhibited had signs of respiratory illness during or shortly after the fair. Ill swine had recovered or had been slaughtered before these interviews. No swine were tested for influenza.

## Discussion

We describe an outbreak of respiratory illness, including 3 confirmed infections with a variant influenza A virus not identified in humans before August 2011. The outbreak occurred during a large agricultural fair, where humans and animals had opportunities for repeated and/or prolonged contact.

Outbreaks of variant influenza A viruses at agricultural events have been described, and these events may be key settings for zoonotic influenza transmission (*17*–*24*). Triple reassortant H3N2 subtype viruses containing the pH1N1 M gene were first identified among swine in the United States in 2009 and have been detected among swine in multiple states, including Pennsylvania (*25*,*26*). During July–November 2011, 13 human infections with A(H3N2)v virus containing the pH1N1 M gene were identified, and 5 were linked to agricultural fairs (*24*). Although the frequency of zoonotic influenza transmission at agricultural events is unknown, these events provide opportunities for swine influenza viruses to infect humans who have contact with infected swine. Human and swine influenza viruses may circulate at these events, creating opportunities for virus reassortment and the emergence of novel strains.

This investigation suggests that swine contact during Fair A was a risk factor for illness. Persons reporting direct contact with swine were more likely to report illness. Most case-patients became ill within 4 days after the swine show or auction, suggesting a temporal relationship between human–swine contact and onset of human illness within <4 days. The epidemic curve, which suggests that case-patients were exposed to a common infectious source that was present for several days, is consistent with the hypothesis that infected swine were present for the duration of the fair. Prior investigations of human variant influenza virus infections have documented contact with infected swine (*17*,*21*), and cases have also occurred after contact with apparently healthy swine (*4*,*23*). No swine were tested for influenza during this investigation because swine at Fair A had either been slaughtered or had recovered before the first human case was reported; however, triple reassortant H3N2 subtype viruses containing genetic material from pH1N1 have been detected in swine (*4*–*6*).

Because of limited diagnostic testing, the extent and distribution of illness caused by A(H3N2)v virus among Fair A attendees are unknown; however, two thirds of children <4 years of age who were tested were seropositive for A(H3N2)v virus. This finding suggests that illness in at least some suspected case-patients can be attributed to A(H3N2)v virus infection. Suspected case-patients had illness onset dates and symptoms similar to those for probable and confirmed case-patients. Symptoms were similar to those of seasonal influenza (*27*), but no seasonal influenza was circulating at the time in Pennsylvania.

Although we cannot rule out human-to-human transmission of A(H3N2)v virus at or after Fair A, enhanced surveillance after Fair A through the beginning of the typical influenza season detected no additional cases of A(H3N2)v virus infection in the community; this suggests that the virus did not exhibit efficient or sustained human-to-human transmission. However, A(H3N2)v virus infection has occurred with limited human-to-human transmission among persons who reported no swine contact (*4*).

This investigation is subject to a number of limitations. First, interviews occurred when media sources began reporting “swine flu” linked to Fair A. Persons who became ill after attending Fair A may therefore have been more likely to report swine exposure, thus biasing toward an association between illness and swine exposure. Second, testing for influenza was not conducted for most case-patients. The timing of the investigation allowed for collection of few respiratory specimens and only convalescent-phase rather than paired serum samples. Serologic testing was further limited to young children because cross-reactive antibodies in older age groups made interpretation of test results for convalescent-phase serum samples difficult. Because only convalescent serum samples were obtained and baseline serologic studies for A(H3N2)v were conducted in a different population, it is possible that elevated HI titers among probable case-patients reflect exposure to A(H3N2)v virus before Fair A. Third, because all members of a household would often attend Fair A together, it was rare to identify ill contacts of case-patients who did not also attend Fair A. This made it difficult to evaluate human-to-human transmission in this population. Fourth, case-patients in the cohort study likely include some persons without A(H3N2)v virus infection, and some persons with mild or asymptomatic A(H3N2)v virus infection may have been considered noncase-patients; the resulting misclassification may have caused underestimation of any association between exposures and illness. One person identified during this investigation had rhinovirus infection identified by rRT-PCR testing, and it is possible that noninfluenza respiratory viruses circulated at Fair A and caused illness in some suspected case-patients. Fifth, the small sample size of the cohort limited our ability to detect statistically significant associations between exposures and case status. Last, we were unable to confirm influenza virus infection among swine at Fair A; therefore, the source of the A(H3N2)v virus cannot be confirmed.

Novel influenza A viruses will continue to emerge sporadically, but steps can be taken to reduce risks to human and animal health. Our findings suggests that swine contact increases risk for A(H3N2)v virus infection; therefore, advising fair attendees, especially those at high risk for complications from influenza, to avoid or limit swine contact may help prevent A(H3N2)v virus infections at agricultural events (*28*). Agricultural club members and others with prolonged swine exposure should also be educated about the risk of zoonotic influenza transmission and actions they can take to reduce transmission risk, such as using personal protective equipment when they or their animals are ill (*29*). We found simultaneous illness in humans and swine at the fair; this finding supports those from prior studies showing that transmission of influenza virus occurs from swine to humans and vice versa (*30*–*32*). Preventing seasonal influenza in humans who have contact with swine (e.g., through annual influenza vaccination) can reduce reassortment opportunities in swine that become co-infected with swine and human influenza viruses. Prompt and thorough investigations should be conducted of all novel influenza virus outbreaks among humans and animals. Investigations can be more timely if patients with influenza-like symptoms inform clinicians of recent swine exposure and if clinicians consider variant influenza virus infection in patients with influenza-like symptoms and recent swine or agricultural fair exposure. Clinicians should work with public health officials to test respiratory specimens by rRT-PCR when they suspect variant influenza virus infection. This investigation was limited by the lack of influenza testing in swine. Representative and timely influenza surveillance among swine, especially during fair season in states where swine are present at agricultural events, would facilitate future investigations.

This outbreak of A(H3N2)v virus infections among persons attending an agricultural fair was likely associated with swine contact. We did not identify sustained human-to-human transmission of A(H3N2)v virus during this investigation; however, the identification of ≈300 human A(H3N2)v virus infections in multiple states during 2011 and 2012 and the occurrence of limited human-to-human transmission in small clusters (*33*,*34*) demonstrate that variant influenza viruses remain a public health concern for animals and humans who may infect each other at venues such as agricultural fairs. Collaboration among public health officials with responsibilities for human and animal health is critical to determining the transmissibility and pandemic potential of variant influenza viruses, such as A(H3N2)v virus, and the epidemiologic features of illnesses caused by them.
